# The Editor's Letter-Box

**Published:** 1919-10-25

**Authors:** F. Murchie

**Affiliations:** For Director-General of Medical Services. The Editor, The Hospital, 28 and 29 Southampton Street, Strand, W.C. 2.


The Editor.

To the Minister of Pensions.

Copy] Ministry of Pensions,
14 Great Smith Street,
Westminster, London, S.W. 1.
October 16, 1919.

Sir,—I am directed by the Minister of Pensions to acknowledge receipt of your letter of yesterday's date and to state that the matter is receiving attention. When a decision has been arrived at a further communication will be addressed to you, but I am to say that if you will be good enough to ask any individual nurses in need of care or treatment for disabilities due to or aggravated by war service to write to The Secretary, M.S.2 (Officers), Ministry of Pensions, 14 Great Smith Street, Westminster, S.W. 1, their claims will be dealt with immediately. I am to say that these nurses should forward some medical evidence of the present condition of their disability and, wherever possible, some evidence of its causation or aggravation by service.—I am, Sir, your obedient servant,

F. Murchie,
For Director-General of Medical Services.

The Editor, The Hospital,
28 and 29 Southampton Street, Strand, W.C. 2.

